# Hybrid Ensemble Deep Learning Model for Advancing Ischemic Brain Stroke Detection and Classification in Clinical Application

**DOI:** 10.3390/jimaging10070160

**Published:** 2024-07-02

**Authors:** Radwan Qasrawi, Ibrahem Qdaih, Omar Daraghmeh, Suliman Thwib, Stephanny Vicuna Polo, Siham Atari, Diala Abu Al-Halawa

**Affiliations:** 1 Department of Computer Science, Al-Quds University, Jerusalem P.O. Box 20002, Palestine; 2 Department of Computer Engineering, Istinye University, Istanbul 34010, Turkey; 3 Department of Medical Imaging, Al-Quds University, Jerusalem P.O. Box 20002, Palestine; ibraheem.qdaih@students.alquds.edu (I.Q.);; 4 Al Quds Business Center for Innovation, Technology, and Entrepreneurship, Al-Quds University, Jerusalem P.O. Box 20002, Palestine; 5 Faculty of Medicine, Al-Quds University, Jerusalem P.O. Box 20002, Palestine

**Keywords:** brain stroke, clinical application, deep learning, hybrid model, image enhancement, images

## Abstract

Ischemic brain strokes are severe medical conditions that occur due to blockages in the brain’s blood flow, often caused by blood clots or artery blockages. Early detection is crucial for effective treatment. This study aims to improve the detection and classification of ischemic brain strokes in clinical settings by introducing a new approach that integrates the stroke precision enhancement, ensemble deep learning, and intelligent lesion detection and segmentation models. The proposed hybrid model was trained and tested using a dataset of 10,000 computed tomography scans. A 25-fold cross-validation technique was employed, while the model’s performance was evaluated using accuracy, precision, recall, and F1 score. The findings indicate significant improvements in accuracy for different stages of stroke images when enhanced using the SPEM model with contrast-limited adaptive histogram equalization set to 4. Specifically, accuracy showed significant improvement (from 0.876 to 0.933) for hyper-acute stroke images; from 0.881 to 0.948 for acute stroke images, from 0.927 to 0.974 for sub-acute stroke images, and from 0.928 to 0.982 for chronic stroke images. Thus, the study shows significant promise for the detection and classification of ischemic brain strokes. Further research is needed to validate its performance on larger datasets and enhance its integration into clinical settings.

## 1. Introduction

Brain strokes, or brain attacks, represent a severe global health problem, significantly contributing to the burden of illness, disability, and mortality worldwide [1,2]. The World Health Organization (WHO) reports that cerebrovascular accidents, commonly known as strokes, are the second leading cause of death and the third leading cause of disability globally [3]. The severity of stroke can vary greatly, from mild cases that resolve with little or no residual disability to severe cases that can lead to death or lifelong dependence on care [4,5]. 

Speedy treatment of stroke is crucial as delays can cause permanent brain damage or increase the severity of the incident on the patient’s healthcare. However, accurately diagnosing strokes quickly in an emergency is difficult due to several factors, including varying levels of expertise among doctors, availability (or lack thereof) of Magnetic resonance imaging (MRI) and Computed tomography (CT) machines for overcrowded hospitals, and a long processing time due to the heavy computational load from processing medical images. These issues can lead to missed or delayed diagnoses and increase the risk of long-term disabilities.

Several methods for ensuring early detection and timely treatment of stroke have been studied. For instance, deep learning, a relatively new yet rapidly developing field of artificial intelligence, has the potential to revolutionize the diagnosis and treatment of stroke. Deep learning models can be trained on large datasets of medical images to learn to identify stroke lesions with high accuracy, even in small or complex cases [6]. These models leverage complex algorithms to extract intelligent, high-dimensional patterns not easily detectable through standard diagnostic procedures. The efficacy of deep learning in stroke diagnosis is gaining attention, primarily due to its proficiency in separating significant features and facilitating differentiation between diverse patient scenarios [5,7,8,9,10,11]. 

In recent years, studies have indicated that deep learning models can outperform human radiologists in detecting and classifying stroke lesions on CT scans and MRI scans [6,7]. Exploring this cutting-edge field, recent research has identified several benchmark deep learning models—including AlexNet, Visual Geometry Group (VGGNet), Inception, and Residual Network (ResNet)—as effective tools in the early detection of brain stroke [12]. 

Employed as transfer learning algorithms, these models have demonstrated considerable success in infarct lesion detection and classification [5,6,7,13]. Within their functionality is an encoder–decoder structure that emphasizes semantic segmentation tasks, empowering these models to precisely identify and categorize stroke-related abnormalities within CT images of the brain [14]. Nevertheless, the effectiveness of these advanced models in automating brain infarction diagnosis is complicatedly connected to the selection of the architectural framework. 

Moreover, researchers have introduced the Hybrid ensemble deep learning (HEDL) models as advanced approaches that combine several deep learning methods, aiming to obtain even better prediction performances [13]. Several studies have used the hybrid model by combining various algorithms, like convolutional neural networks (CNNs), recurrent neural networks (RNNs), and long short-term memory networks (LSTM), all with varying results [15,16,17]. 

Despite promising results, machine learning faces several significant challenges in detecting early-stage ischemic strokes. One major issue is the quality and variety of data. Medical imaging data can be inconsistent due to differences in equipment, scanning protocols, and patient conditions. Limited labeled data is another critical challenge. Annotating medical images requires expert knowledge, making it difficult to amass large datasets for training machine learning models.

The subtle and varied nature of stroke features further complicates detection. Early-stage ischemic strokes often present with minute changes in the brain that can be easily overlooked. These features can vary widely between patients, necessitating robust models that can generalize across diverse cases. Additionally, the inherent complexity of medical images, with their high-dimensional and noisy nature, poses a significant obstacle in accurately identifying stroke-related abnormalities.

Extracting important features from these images and making models that perform well in different settings is also challenging. Feature extraction requires sophisticated techniques to capture relevant information without losing critical details. Ensuring that these models work effectively across various clinical environments, with different imaging devices and patient demographics, adds another layer of difficulty.

Considering the above, this study seeks to introduce a novel approach for speedy detection of stroke in emergency situations by combining advanced image processing techniques with a unique deep learning network. The image processing techniques simplify and classify ischemic strokes more accurately, overcoming previous obstacles and improving diagnosis reliability. This technological advance not only boosts diagnostic precision in a shorter time but also integrates smoothly into clinical workflows, enhancing patient care and reducing the chances of long-term after-effects from strokes.

## 2. Materials and Methods

This study utilized both CT and MRI scans from Istishari Arab Hospital (IAH) to capture different stroke stages, including normal, acute, chronic, hyper-acute, and sub-acute. The equipment used includes the Philips Incisive scanner and the Philips Ingenia MRI scanner (Philips Healthcare, Amsterdam, Netherlands).

The brain CT scans were performed using the Philips Incisive scanner (128 slices) at IAH. The technical specifications for the CT scans were a tube current of 300 mA, a peak voltage of 120 kVp, a window width/level (WW/WL) of 80/40 HU, a matrix size of 512 × 512, a slice thickness of 0.625 mm, and a reconstruction technique utilizing iDose 3.

For the diffusion-weighted MRI (DW-MRI) sequences, the Philips Ingenia 1.5 T MRI scanner at IAH was employed. The technical specifications for the DW-MRI sequences included an echo time (TE) of 85 ms, a repetition time (TR) of 4000 ms, a field of view (FOV) of 230 × 230 mm, a matrix size of 152 × 106, and a b-Factor of 0 & 1000.

Neurologists and radiologists classified the strokes into hyper-acute, acute, sub-acute, and chronic categories, following recognized medical standards. Each classification was verified by clinical diagnosis and follow-up. The research protocol was approved by the Al-Quds University Institutional Review Board (IRB), under code 410/REC/2023.

### 2.1. Dataset 

In 2023, our study collected a dataset of 10,000 images from public hospitals across Palestine, featuring five categories: normal, hyper-acute, acute, sub-acute, and chronic, with 2000 cases each. The images, standardized to 640 × 640 pixels and JPEG format, trained our advanced diagnostic model.

The dataset was divided into four training groups: Normal-Hyper-acute, Normal-Acute, Normal-Sub-acute, and Normal-Chronic. To enhance diversity and prevent overfitting, we applied data augmentation techniques, quadrupling the dataset size. The augmented dataset was split into training (70%), testing (20%), and validation (10%) segments, using 25-fold cross-validation and dropout layers. Additionally, a supplementary set of 500 real-world clinical images validated the model’s accuracy and clinical relevance.

### 2.2. Hybrid Ensemble Deep Learning for Clinical Stroke Diagnosis 

This study introduces a novel HEDL model for diagnosing and classifying ischemic brain strokes in CT medical images. The model has three main phases: (1) medical image enhancement and data preparation, using the Software Process Engineering Metamodel (SPEM) model to improve image quality and clarity for clinical stroke diagnosis; (2) the HEDL model, combining Dense Convolutional Network with 121 layers (DenseNet121) and Ensemble Deep Random Vector Functional Link (edRVFL) algorithms for their effectiveness with stroke image datasets; and (3) a novel approach for stroke lesion detection and segmentation, utilizing Medical Segmentation Anything Model (MedSAM) and the “You Only Look Once version 5” (YOLOv5) object detection model [18,19,20]. 

**Figure 1:**
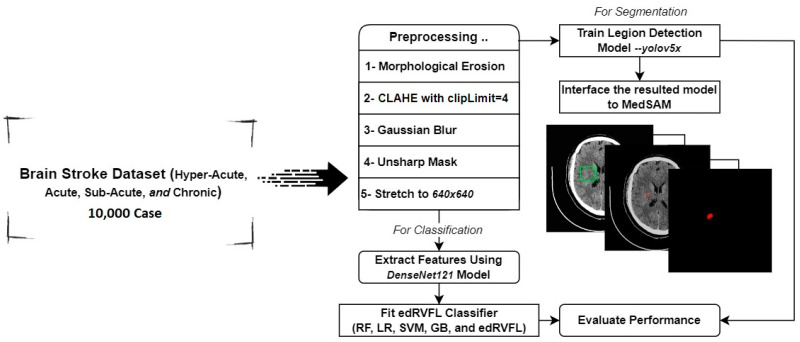
Architecture of the model.

We employed transfer learning to adapt the Visual Geometry Group 16-layer model (VGG16) and DenseNet121 architectures within the CNN to our stroke image dataset. A comparative analysis of both architectures helped identify the most effective model for integration with the edRVFL outputs, creating a robust hybrid deep learning framework. Figure 1 illustrates the architecture of the model, highlighting the integration process and the flow of data through the different components.

#### 2.2.1. Stroke Precision Enhancement Model (SPEM)

The study introduced the stroke precision enhancement model (SPEM) as an approach for enhancing CT image quality to aid in stroke prediction through deep learning analysis. SPEM employs morphological erosion to reduce noise and simplify raw CT images, enhancing visibility for stroke diagnosis [21]. It then uses contrast-limited adaptive histogram equalization (CLAHE) to redistribute pixel intensity values, enhancing contrast crucial for accurate stroke prediction [22]. The effectiveness of CLAHE in SPEM is evaluated using the effective measure of enhancement (EME) and peak signal-to-noise ratio (PSNR), ensuring that SPEM enhances images while maintaining the integrity and accuracy needed for reliable stroke prediction.

The following equation is utilized for EME:

(1) $$EME=\frac{1}{K_{1}K_{2}}\sum_{L=1}^{K_{2}}\sum_{K=1}^{K_{1}}20\;log\left(\frac{I_{max}\left(k,l\right)}{I_{min}\left(k,l\right)}\right)$$

where $K_{1},K_{2}$ are the number of horizontal and vertical blocks in the image, $I_{max}\left(k,l\right)$, and $I_{min}\left(k,l\right)$ are the maximum and minimum pixel values in each block.

The peak signal-to-noise ratio (PSNR) was used to measure the deviation of the current image from the original image with respect to the peak value of the gray level. Given a reference image f and a test image g, both of size M×N, the PSNR between f and g is defined by:

(2) $$PSNR(f,g)=10\left(255^{2}/MSE(f,g)\right)$$

where

(3) $$MSE(f,g)=\frac{1}{MN}\sum_{i=1}^{M}\sum_{j=1}^{N}{\left(f_{ij}-g_{ij}\right)}^{2}$$

In SPEM, as the Mean Squared Error (MSE) approaches zero, the PSNR increases, indicating improved image quality. A higher PSNR means the enhanced image closely resembles the original CT scan, retaining essential details for accurate stroke prediction.

SPEM also uses the Laplacian of Gaussian (LoG) edge enhancement technique, which begins with Gaussian smoothing followed by the application of a Laplacian operator to highlight edges of pathological structures, crucial for stroke analysis [23]. Furthermore, SPEM employs unsharp masking to sharpen CT image details. The masking technique involves creating a blurred, or “unsharp”, version of the image and then subtracting it from the original. The resulting high-contrast mask is then added back to the original image. This step enhances the sharpness and clarity of the image without introducing unwanted noise or artifacts, maintaining the integrity of diagnostic details [24].

#### 2.2.2. Adaptation of Ensemble Deep Random Vector Functional Link for Stroke Classification

In the context of our study, the ensemble deep random vector functional link (edRVFL) has been adapted for classifying strokes, which integrates deep and ensemble learning techniques, furthering the random vector functional link (RVFL) network’s legacy. Initially brought forward by Hu et al. (2023) and built upon the single-layer feedforward network introduced by Zhang et al. (2016), RVFL is praised for its straightforward design which is remarkably capable in several machine learning models [25,26,27].

The RVFL network is known for its effectiveness in machine learning, image classification, pattern recognition, and signal processing. It has a simple architecture with three layers:

1. Input layer: processes incoming data.
2. Hidden layer: uses randomly initialized weights for unbiased data processing.
3. Output layer: produces the final output.

During training, the focus is on adjusting the weights of the output layer, often by solving a linear least squares problem. Methods like the conjugate gradient method can improve this process’s efficiency.

In the RVFL, features from each model level are merged with the original input data after undergoing nonlinear transformations, enhancing the model’s complexity and effectiveness. The core computation of the RVFL is outlined by a specific mathematical formula that describes this process of feature integration and transformation:

(4) $$y=W_{o}\;tanh\;tanh\;\left(W_{i}x+b_{i}\right)+b_{o\;}$$

wherein:

- y stands for the predicted output.
- W_o_ and W_i_ designate the weights associated with the output and hidden layers, respectively.
- b_i_ and b_o_ are the bias terms corresponding to the hidden and output layers.
- x is representative of the input data.

**Figure 2:**
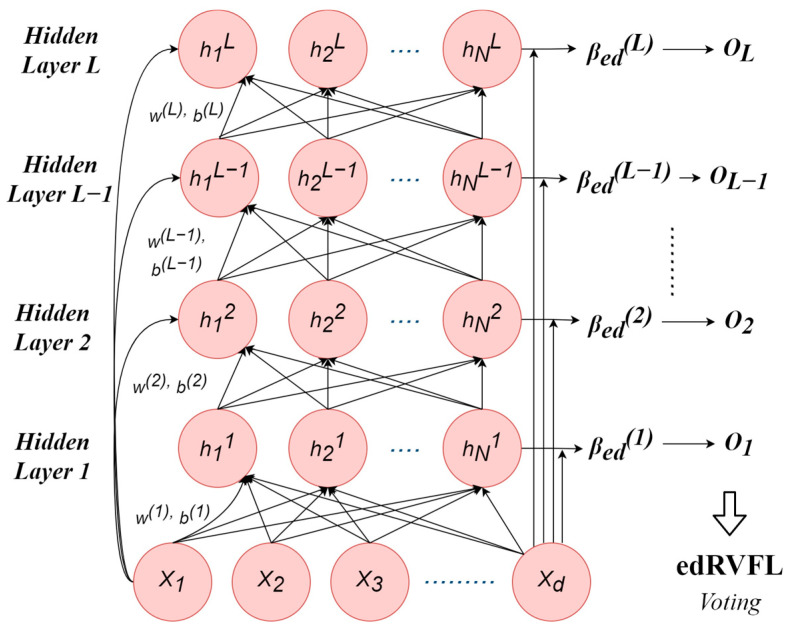
The ensemble deep RVFL neural network architecture.

The edRVFL approach improves upon the basic RVFL by creating an ensemble of multiple RVFL networks, each trained on different segments of the dataset. This ensures that each network specializes in a unique subset of data. The outputs from these networks are then combined to form a more robust model. This integration results in reduced overfitting and improved generalization across diverse datasets. The mathematical framework of the edRVFL reflects this enhanced approach (see Figure 2):

(5) $$edRVFL\left(x\right)=F\left(XW_{1}+b_{1}\right)W_{2}+b_{2\;}$$

For clarity:

- x symbolizes the input data.
- W_1_ and W_2_ represent weight matrices corresponding to the sequential RVFL networks.
- b_1_ and b_2_ denote bias vectors that are aligned with the respective RVFL networks.
- F is indicative of a nonlinear activation function.

The training process for the edRVFL involves several key steps:

- Begin by randomly assigning the weight matrices and bias vectors in the RVFL networks.
- Conduct individual training sessions for each RVFL network, tailoring them to specific data subsets for the best results.
- Combine the outputs from all RVFL networks to create a unified edRVFL output.
- Repeatedly adjust and fine-tune the process by revisiting steps 2 and 3 until the model reaches optimal performance.

#### 2.2.3. Enhancing Stroke Detection by Advancing edRVFL Classification Capabilities

Our research has refined the edRVFL algorithm parameters to better classify strokes, emphasizing early detection of acute events. We have enhanced the algorithm’s efficiency and accuracy in stroke classification, focusing on early-stage identification, reducing execution times, and minimizing reliance on conventional feature engineering.

##### In-Depth Hyperparameter Optimization

Our research focuses on optimizing edRVFL’s hyperparameters for better stroke classification. We have identified three key hyperparameters: enhancement node count, regularization parameter, and hidden layer quantity. Their precise calibration balances algorithm performance and resource efficiency.

We adjust enhancement nodes for optimal classification and manage the computational load. The regularization parameter is fine-tuned to prevent overfitting and ensure memory efficiency. The optimal number of hidden layers is crucial for handling complex data features while considering computational demands and execution speed.

The optimal hyperparameter values found are:

1. Enhancement nodes: 10
2. Regularization parameter: 100
3. Hidden layers: 10

##### Enhancing Feature Extraction and Clustering Techniques

We have enhanced the edRVFL framework by integrating DenseNet 121, a state-of-the-art deep learning architecture, improving its ability to detect complex patterns in stroke images and simplifying classification. This approach outperforms standard feature engineering in biological classification.

Our method surpasses traditional clustering techniques by leveraging the hierarchical structure of data and capturing subtle image relationships. We used a 25-fold cross-validation technique in the clustering process to ensure the robustness and reliability of our results. This is crucial for analyzing our curated dataset of 500 stroke cases, augmented for increased robustness and diversity. Our hierarchical clustering strategy achieves more precise and relevant grouping outcomes by accurately reflecting the nuanced relationships between images.

#### 2.2.4. Ablation Study 

In our study, we conducted an ablation study to dissect the contribution of individual components within our proposed ensemble model for diagnosing and classifying ischemic brain strokes in CT medical images. The baseline ensemble model consists of a combination of customized CNN, VGG16, and DenseNet121 architectures.

To assess the impact of each component within the ensemble model, we conducted a series of experiments wherein we systematically removed one model from the ensemble at a time. For instance, we eliminated the CNN and observed the resultant effects on performance metrics such as accuracy, precision, recall, F1 score, and AUC (area under the curve). The subsequent configurations after the elimination of one model include the ensembles of CNN + VGG16, CNN + DenseNet121, and VGG16 + DenseNet121, as well as individual models such as CNN, VGG16, and DenseNet121.

Objective measures, including accuracy, precision, recall, F1 score, and AUC, are employed to quantitatively evaluate the effectiveness of the model in enhancing stroke diagnosis accuracy.

#### 2.2.5. Developing a Novel Method for Lesion Detection and Segmentation in Medical Imaging

Quantitative analysis in medical imaging relies heavily on segmentation, but traditional methods are limited by the need for extensive manual data labeling. Foundation models like Meta’s Segment Anything Model (SAM) by Alexander Kirillov et al. in 2023, and MedSAM by Bo Wang et al. in 2023, show promise in overcoming these limitations by pre-training on diverse datasets [18]. SAM provides broad segmentation capabilities across imaging types using text prompts. MedSAM, trained on over one million image–mask pairs, excels in medical segmentation, outperforming specialist models in some cases due to its superior generalization [19]. Furthermore, in our study, the YOLOv5 object detection model on the dataset was employed [20]. YOLOv5 was chosen for its exceptional performance in real-time object detection. It was trained for 250 epochs using the stochastic gradient descent (SGD) optimizer, with an initial learning rate of 0.001, and common data augmentation techniques like flipping, rotations, and cropping.

Our primary metric for segmentation accuracy was the Dice similarity coefficient (DSC), a widely used metric in medical imaging for evaluating segmentation performance. It measures the spatial overlap between two segmentation masks on a scale from 0 to 1, with a score of 1 indicating perfect overlap. The formula for calculating the DSC is:

(6) $$DSC=\frac{2\;|A\;\cap \;B|}{\left|A|+|B\right|}$$

A: Predicted Segmentation 

B: True Segmentation

### 2.3. Model Validation

We evaluated various machine learning models for stroke prediction on a clinical dataset of 500 CT brain scans, comparing results with actual diagnoses. The evaluation used 25-fold cross-validation and metrics like accuracy, precision, recall, F1 score, and AUC to assess consistency and generalization, identifying the most effective algorithm for stroke detection.

The hybrid approach combines supervised detection and unsupervised segmentation to reduce manual annotation dependency. We employed YOLOv5 for real-time detection efficiency, achieving high accuracy in lesion detection on annotated scans from Palestinian hospitals. MedSAM was then used for precise lesion segmentation, guided by YOLOv5’s detections. This method leverages the strengths of both models, offering a robust solution for accurate medical image segmentation.

## 3. Results

### 3.1. Hybrid Model Performance before Enhancement

Table 1’s analysis reveals the performance of various machine learning classifiers on an original brain ischemic stroke dataset before integrating the SPEM model. For hyper-acute strokes, SVM led in accuracy (94.9%), closely followed by random forest (92.0%), with random forest (41.87 s) being quicker than SVM (53.05 s). In acute strokes, edRVFL was most accurate (95.4%), with logistic regression nearly matching (95.3%). Nonetheless, LR was faster in processing time than the edRVFL at 0.06 s and 1.01 s, respectively. Sub-acute stroke detection saw SVM and edRVFL both achieving 96.3% accuracy, with SVM faster at 0.34 s compared to edRVFL’s 0.45 s. For chronic strokes, SVM and edRVFL impressed with 97.5% and 97.9% accuracies, respectively, and were efficient in time, with SVM at 0.61 s and edRVFL at 0.74 s. Overall, SVM consistently offered high accuracy and time-efficiency across stroke types, with logistic regression and edRVFL also showing strong performance in both accuracy and speed.

### 3.2. Hybrid Model Performance with SPEM Image Enhancement Top of Form

Table 2 reveals the impact of the SPEM model alongside DenseNet121 on enhancing machine learning classifiers’ performance on an enhanced dataset. In hyper-acute stroke detection, edRVFL and logistic regression led with accuracies of 0.9958 and 0.9957, respectively, combining high precision with low processing times of 0.74 and 0.04 s. SVM also showed strong performance with a 0.9915 accuracy and 0.52-s processing time, while random forest, despite its lower accuracy of 0.9832, completed predictions in 3.30 s.

For acute strokes, SVM, logistic regression, and edRVFL classifiers all recorded accuracies above 0.99. SVM’s accuracy stood at 0.9930, requiring 24.74 s, whereas logistic regression showed a near-identical accuracy of 0.9922 but with a much quicker 0.42 s processing time. edRVFL achieved the highest accuracy at 0.9933, with a processing time of 2.46 s. Random Forest lagged slightly in accuracy (0.9716) and took longer at 42.73 s.

In sub-acute stroke analysis, edRVFL had the top accuracy of 0.9816, with a quick 0.45 s processing time. SVM and logistic regression both notched accuracies of 0.975, with logistic regression being the fastest at 0.03 s, slightly edging out SVM’s 0.31 s. Random Forest was less accurate (0.9509) but maintained efficiency with a 2.07 s processing time.

In chronic stroke analysis, classifier performance notably improved. The edRVFL classifier achieved an impressive accuracy of 0.9980 with a processing time of 1.02 s. SVM and logistic regression both recorded accuracies of 0.9959, with SVM slightly less time-efficient at 1.83 s, compared to logistic regression’s swift 0.06 s. Random Forest, although the least accurate at 0.9736, maintained a quick processing time of 8.36 s.

### 3.3. Insights from Ablation Study

The results of the ablation study, as summarized in Table 3, provide valuable insights into the significance of each model within the ensemble for stroke diagnosis.

The results indicate that DenseNet121 plays a crucial role in contributing to the overall performance metrics of the ensemble model. DenseNet121 significantly enhances the final performance when included in the ensemble, as evidenced by the high accuracy scores achieved in both the hyper-acute and acute stages. In particular, DenseNet121 achieved the highest accuracy scores of 0.9958 and 0.9933 for the hyper-acute and acute stages, respectively, surpassing the performance of the baseline model.

Moreover, removing DenseNet121 from the ensemble resulted in a significant decrease in performance across all stages, highlighting its substantial impact on the overall effectiveness of the model.

However, while the customized CNN architecture achieves the best result for the sub-acute stage, it does not perform as well as DenseNet121 in other stages. Interestingly, the combination of CNN and DenseNet121 in the ensemble resulted in the highest accuracy for the chronic stage, showcasing the complementary strengths of these two architectures when used together.

Based on these findings, only DenseNet121 was used in our proposed architecture. This decision is motivated by its superior performance across different stages and the desire to streamline the model architecture, thus avoiding unnecessary computational overhead and reducing processing time.

It is worth noting that all experiments were conducted on enhanced datasets utilizing contrast limited adaptive histogram equalization (CLAHE) with a clip limit of 4. Additionally, the final classification was performed using the enhanced discriminative random vector functional link (edRVFL) algorithm, ensuring robustness and accuracy in the diagnosis of ischemic brain strokes.

### 3.4. Hybrid Model’s Validation in Clinical Setting

Table 4 presents a comparison of hierarchical clustering’s performance on raw versus SPEM-enhanced stroke images using the edRVFL model. For hyper-acute stroke images, accuracy increased from 0.876 in the raw dataset to 0.933 with SPEM enhancement, demonstrating clear accuracy benefits for hyper-acute cases. In acute stroke images, accuracy improved from 0.881 with no enhancement to 0.948 post-SPEM application, underscoring the value of image enhancement in acute stroke classification.

Sub-acute stroke images saw a marked accuracy increase from 0.927 in the original to 0.974 with SPEM enhancement, indicating the technique’s effectiveness in sub-acute cases. Chronic stroke images experienced the most significant accuracy boost, from 0.928 in the raw dataset to 0.982 with enhancement, highlighting SPEM’s role in accurately classifying chronic strokes.

### 3.5. Hybrid Segmentation Model 

Results in Table 5 indicated the success of a hybrid segmentation method combining MEDSAM and YOLOv5, validated with clinical data from the Palestinian healthcare system. The results indicated the hybrid model’s segmentation efficiency using Dice similarity coefficients (DSCs), comparing original and SPEM-enhanced data. Original data showed moderate to high segmentation efficiency across stroke types, with DSCs ranging from 0.7 to 0.96, the highest being for chronic strokes. SPEM enhancement significantly improved DSCs for acute, hyper-acute, and sub-acute strokes to 0.91, 0.92, and 0.98 respectively. 

**Figure 3:**
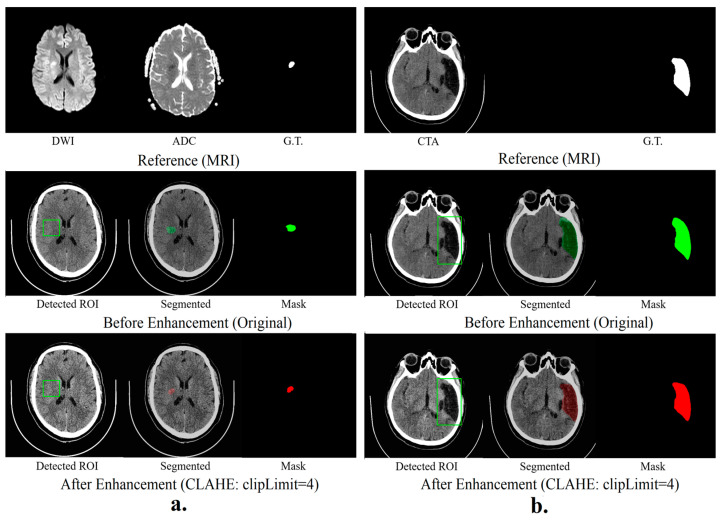
Hybrid segmentation models (**a**) show the results of hyper-acute segmentation, and (**b**) show the chronic segmentation results.

Results in Figure 3 reveal that the hybrid segmentation method, especially with SPEM-enhanced data, excels in accurately identifying stroke lesions in CT scans. The noticeable boost in Dice similarity coefficients (DSCs) for hyper-acute, acute, and sub-acute strokes highlights the significant role of image enhancement in increasing lesion detection accuracy for these stroke types. On the other hand, for chronic strokes, the unenhanced original data yielded marginally superior outcomes, indicating that SPEM’s effectiveness may differ based on the stroke’s nature and features.

## 4. Discussion

This study successfully demonstrates the potential of a HEDL model, empowered by image enhancement, for improving brain stroke detection and classification in clinical applications. This model leverages the complementary strengths of diverse architectures, notably DenseNet 121 and ensemble deep random vector functional link (edRVFL) networks, to achieve high accuracy across different stroke types. Furthermore, the integration of the SPEM image enhancement technique significantly elevates the model’s performance, particularly for hyper-acute and acute stroke types. These findings align with previous research highlighting the efficacy of hybrid deep learning approaches and image preprocessing for enhancing medical image analysis for diagnoses and classification [16,17,28].

The synergy within the hybrid model lies in the strategic combination of distinct strengths. edRVFL networks, recognized for their robustness and interpretability in medical applications [15,16], provide a reliable foundation for accurate stroke classification. DenseNet121, known for its exceptional efficiency and speed [17], complements edRVFL by ensuring rapid processing, a crucial factor for real-world clinical applications. This combination empowers the model to deliver high accuracy while maintaining practical feasibility. These findings align with other studies indicating the efficiency and robustness of DenseNet121 in medical diagnosis and object recognition [29,30]. 

Secondly, this study emphasizes the remarkable impact of image enhancement on model performance. The SPEM model, by enhancing image clarity and highlighting stroke-related abnormalities, significantly boosts the accuracy of all classifiers across diverse stroke types. This finding corroborates previous research advocating for the importance of image preprocessing and enhancement in boosting the accuracy and reliability of deep learning models in medical image analysis [5,6,8,31]. Notably, the accuracy improvement was most noticeable for hyper-acute, acute, and sub-acute stroke cases, suggesting that image enhancement may be particularly beneficial for these challenging categories.

The hybrid segmentation model, integrating MedSAM and YOLOv5, further strengthens the model’s capabilities by enabling accurate lesion detection and visualization. This approach, combining the object detection ability of YOLOv5 with MEDSAM’s lesion segmentation expertise, achieved high accuracy in identifying stroke lesions, particularly in acute and hyper-acute cases. This aligns with prior studies demonstrating the effectiveness of hybrid segmentation models for medical image analysis tasks [9,14,32]. 

Therefore, this study demonstrates the efficacy of a hybrid deep learning model with image processing for superior stroke detection and classification in clinical settings. The model’s high accuracy and operational efficiency, particularly through the hybrid prediction method, indicate its readiness for clinical use. The SPEM image enhancement technique boosts the precision of classifiers in identifying ischemic strokes, especially in hyper-acute and acute cases. 

The integration of MEDSAM and YOLOv5 further improves accuracy, especially in acute and hyper-acute strokes.

## 5. Limitations

Despite these promising results, some limitations necessitate further investigation. First, the model requires validation and testing on larger and more diverse datasets to ensure generalizability and robustness. Second, enhancing the model’s interpretability would provide valuable insights into its decision-making process and build trust among clinicians. Finally, seamless integration with existing clinical workflows is crucial for successful real-world implementation. Addressing these limitations will promote the real-world deployment of this model, enabling more accurate and efficient brain stroke diagnosis and management, and thus better care and quality of life for stroke patients. 

## 6. Conclusions

This study highlights the potential of a hybrid ensemble deep learning model with image enhancement for improved brain stroke detection and classification in clinical applications. The model’s high accuracy and efficiency, coupled with the effectiveness of the hybrid segmentation approach, suggests its potential for clinical adoption. The study demonstrates the effectiveness of the SPEM image enhancement technique in significantly improving the accuracy of machine learning classifiers for predicting ischemic strokes. Before enhancement, SVM, logistic regression, and edRVFL showed promise in accuracy and processing times. However, after enhancement, accuracy levels notably increased, particularly for hyper-acute and acute strokes. edRVFL and logistic regression consistently achieved high accuracy with low processing times, making them practical choices. SVM maintained high accuracy but required a slightly longer processing time, while random forest offered a balance between accuracy and speed. These results emphasize the potential of image enhancement methods for enhancing stroke prediction models and improving early diagnosis and treatment. This study encourages further research in this field to benefit healthcare professionals and patient outcomes.

## Figures and Tables

**Table 1:** Performance evaluation of the original data using different machine learning classifiers, concerning the DenseNet121 Deep Learning Model.

Brain Ischemic Stroke Prediction							
Cases	Classifier	Accuracy	Precision	Recall	F1	AUC	Time (s)
Hyper-acute	Random Forest	0.920	0.904	0.926	0.915	0.975	41.87
	SVM	0.949	0.940	0.952	0.946	0.989	53.05
	Logistic Regression	0.908	0.907	0.895	0.901	0.967	0.4
	edRVFL	0.910	0.910	0.910	0.910	0.972	2.4
Acute	Random Forest	0.907	0.921	0.927	0.924	0.968	8.63
	SVM	0.939	0.963	0.937	0.950	0.987	2.52
	Logistic Regression	0.953	0.963	0.960	0.962	0.988	0.06
	edRVFL	0.954	0.948	0.947	0.947	0.990	1.01
Sub-acute	Random Forest	0.933	0.932	0.919	0.925	0.992	2.35
	SVM	0.963	0.986	0.932	0.958	0.992	0.34
	Logistic Regression	0.957	0.947	0.960	0.953	0.994	0.04
	edRVFL	0.963	0.963	0.963	0.963	0.996	0.45
Chronic	Random Forest	0.945	0.950	0.943	0.947	0.986	3.44
	SVM	0.975	0.983	0.967	0.975	0.998	0.61
	Logistic Regression	0.978	0.988	0.959	0.979	0.999	0.05
	edRVFL	0.979	0.980	0.979	0.979	0.999	0.74

**Table 2:** Performance evaluation of the enhanced data using the SPEM model, using different machine learning classifiers, concerning the DenseNet121 Deep Learning Model.

Enhanced Dataset at CLAHE (Clip Limit = 4)							
Cases	Classifier	Accuracy	Precision	Recall	F1	AUC	Time (s)
Hyper-acute	Random Forest	0.9832	0.9918	0.976	0.9838	0.9995	3.30
	SVM	0.9915	0.9830	0.9945	0.9915	0.9998	0.52
	Logistic Regression	0.9957	0.9914	0.9932	0.9957	0.9999	0.04
	edRVFL	0.9958	0.9957	0.9958	0.9958	0.9999	0.74
Acute	Random Forest	0.9716	0.9534	0.9896	0.9711	0.9961	42.73
	SVM	0.9930	0.9931	0.9930	0.9930	0.9997	24.74
	Logistic Regression	0.9922	0.9919	0.9919	0.9919	0.9994	0.42
	edRVFL	0.9933	0.9933	0.9933	0.9933	0.9994	2.46
Sub-acute	Random Forest	0.9509	0.9398	0.9630	0.9512	0.9918	2.07
	SVM	0.9754	0.9639	0.9876	0.9756	0.9993	0.31
	Logistic Regression	0.9755	0.9753	0.9753	0.9753	0.9986	0.03
	edRVFL	0.9816	0.9817	0.9816	0.9817	0.9988	0.45
Chronic	Random Forest	0.9736	0.9649	0.9892	0.9769	0.9980	8.36
	SVM	0.9959	0.9928	0.9941	0.9964	0.9999	1.83
	Logistic Regression	0.9959	0.9928	0.9976	0.9964	0.9997	0.06
	edRVFL	0.9980	0.9980	0.9980	0.9980	0.9999	1.02

**Table 3:** Comparative ablation study results for stroke diagnosis model configurations.

Cases	Experiment	Accuracy	Precision	Recall	F1 Score	AUC
Hyper-acute	Baseline Model	0.9923	0.9922	0.9923	0.9923	0.9998
	Ensemble of CNN, VGG16	0.9906	0.9905	0.9906	0.9906	0.9996
	Ensemble of CNN, DenseNet	0.9936	0.9935	0.9936	0.9936	0.9999
	Ensemble of VGG16, DenseNet	0.9927	0.9926	0.9928	0.9927	0.9998
	CNN	0.9912	0.9911	0.9911	0.9911	0.9998
	VGG16	0.9898	0.9896	0.9898	0.9897	0.9996
	DenseNet121	0.9958	0.9957	0.9958	0.9958	0.9999
Acute	Baseline Model	0.9925	0.9926	0.9925	0.9925	0.9994
	Ensemble of CNN, VGG16	0.9924	0.9925	0.9925	0.9925	0.9992
	Ensemble of CNN, DenseNet	0.9932	0.9931	0.9931	0.9931	0.9994
	Ensemble of VGG16, DenseNet	0.9926	0.9925	0.9926	0.9926	0.9993
	CNN	0.9928	0.9929	0.9929	0.9929	0.9994
	VGG16	0.9922	0.9921	0.9921	0.9921	0.9992
	DenseNet121	0.9933	0.9933	0.9933	0.9933	0.9994
Sub-acute	Baseline Model	0.9855	0.9854	0.9854	0.9854	0.9988
	Ensemble of CNN, VGG16	0.9871	0.9871	0.9872	0.9872	0.9989
	Ensemble of CNN, DenseNet	0.9878	0.9877	0.9876	0.9877	0.9991
	Ensemble of VGG16, DenseNet	0.9872	0.9871	0.9871	0.9871	0.9987
	CNN	0.9932	0.9934	0.9933	0.9933	0.9991
	VGG16	0.9811	0.9812	0.9811	0.9811	0.9989
	DenseNet121	0.9816	0.9817	0.9816	0.9817	0.9988
Chronic	Baseline Model	0.9961	0.9962	0.9963	0.9963	0.9998
	Ensemble of CNN, VGG16	0.9952	0.9951	0.9951	0.9951	0.9996
	Ensemble of CNN, DenseNet	0.9981	0.9982	0.9981	0.9981	0.9998
	Ensemble of VGG16, DenseNet	0.9975	0.9976	0.9976	0.9976	0.9997
	CNN	0.9978	0.9979	0.9978	0.9978	0.9998
	VGG16	0.9916	0.9915	0.9915	0.9915	0.9993
	DenseNet121	0.9980	0.9980	0.9980	0.9980	0.9999

**Table 4:** Comparative performance evaluation of hierarchical clustering on raw stroke images and images enhanced with the use of hybrid edRVFL model.

	Accuracy				Time (s)
	Hyper-Acute	Acute	Sub-Acute	Chronic	
Original without enhancement	0.876	0.881	0.927	0.928	0.2922
SPEM at CLAHE = 4	0.933	0.948	0.974	0.982	0.2550

**Table 5:** The efficiency of the hybrid segmentation model based on Dice similarity coefficients (DSCs) estimation compared to MedSAM.

Stroke Level	Dice Similarity Coefficient (MedSAM)	Dice Similarity Coefficient (SPEM)
Acute	0.7	0.91
Hyper-acute	0.71	0.92
Sub-acute	0.8	0.92
Chronic	0.96	0.98

## Data Availability

The original data presented in the study are available on request.
